# Associations of Body Mass Index and Percent Body Fat with Osteoporosis, Sarcopenia, and Osteosarcopenia: A Retrospective Study Based on Postmenopausal Women in China

**DOI:** 10.3390/healthcare13010028

**Published:** 2024-12-26

**Authors:** Shengli Zhao, Jiacong Hong, Haonan Li, Xiaoyan Zhang, Yong Wan, Bailing Chen

**Affiliations:** 1 Department of Spine Surgery, The First Affiliated Hospital of Sun Yat-sen University, Guangzhou 510080, China; zhaoshli6@mail.sysu.edu.cn (S.Z.); hongjc@mail3.sysu.edu.cn (J.H.); lihn5@mail3.sysu.edu.cn (H.L.); zhangxy359@mail.sysu.edu.cn (X.Z.); 2 Department of Nutrition, School of Public Health, Sun Yat-sen University, Guangzhou 510080, China

**Keywords:** body mass index, percent body fat, osteoporosis, sarcopenia, osteosarcopenia

## Abstract

**Background/Objectives**: Alterations in the body mass index (BMI) and percent body fat (PBF) have been considered to be related to aging-induced changes in bone and muscle. This study aimed to evaluate the associations of the BMI and PBF with osteoporosis, sarcopenia, and osteosarcopenia in postmenopausal women. **Methods**: A total of 342 participants who underwent musculoskeletal function assessments at the First Affiliated Hospital of Sun Yat-sen University between January 2015 and December 2022 were retrospectively screened. The diagnosis of osteoporosis was based on the WHO criteria, and sarcopenia was diagnosed according to the 2019 consensus of the Asian Working Group for Sarcopenia. **Results**: The BMI was positively correlated with the musculoskeletal function assessment parameters (bone mineral density, relative skeletal muscle index, and grip strength) and identified as an independent protective factor for sarcopenia alone (OR = 0.592, 95% CI: 0.455–0.769) or osteosarcopenia (OR = 0.411, 95% CI: 0.319–0.529), with a moderate diagnostic accuracy (area under the curve [AUC] = 0.682) for the former and a high diagnostic accuracy (AUC = 0.823) for the latter. However, the PBF was negatively correlated with the relative skeletal muscle index and identified as a risk factor for osteosarcopenia (OR = 1.404, 95% CI: 1.007–1.959), with a moderate diagnostic accuracy (AUC = 0.613). **Conclusions**: A higher BMI and lower PBF were associated with a lower prevalence of osteosarcopenia in postmenopausal women. Further research is required to elucidate the independent effects of the BMI and PBF on bone health.

## 1. Introduction

Osteoporosis (OP) and sarcopenia (SP) are common clinical manifestations of a deteriorating musculoskeletal system, and both are associated with an increased risk of mortality and premature death in the elderly [1]. Osteosarcopenia (OS) has recently emerged as a new concept indicating the co-existing states of bone loss and muscle degeneration [2]. The prevalence of OS ranges from 1.7% to 34.0%, and it is especially prevalent in the frail population [3,4,5]. In the results of some large clinical studies, the rate of serious adverse events of OS is considerably higher than the sum of risks contributed by OP or SP alone [1,6]. Postmenopausal women are particularly vulnerable to OS. A recent cross-sectional study conducted in Finland revealed a significant decline in both bone mass and muscle quality following menopause [7]. Therefore, it is necessary to effectively distinguish OP, SP, and OS in postmenopausal populations given their different risks of adverse clinical outcomes.

The body mass index (BMI) is now the most widely used measure of adiposity globally. In a large-scale cohort study, overweight and obesity states, as defined by BMI, were associated with higher frailty levels both cross-sectionally and longitudinally [8]. A study in adults living in nursing homes found that a low BMI was a predictor of SP, and this conclusion was supported by a study from Choi et al. [9,10]. Zheng et al. also highlighted the importance of avoiding a low baseline BMI, prior BMI loss, and high baseline central obesity to prevent osteoporotic fractures [11].

Percent body fat (PBF) is another important indicator of health that can be quickly obtained in an outpatient setting using a bioelectrical impedance method. Excess adiposity measured as PBF correlates well with an increased risk of cardiovascular disease, diabetes, and other obesity-associated comorbidities [12]. In addition, PBF was reported to be negatively correlated with the tibial cortical area and bone mineral density (BMD) [13,14,15]. Jayaraman et al.’s study confirmed an independent positive association between PBF and frailty and indicated that PBF may mediate the association between the BMI and frailty [8]. The close relationship between body fat and metabolism makes PBF a potentially valuable indicator for evaluating musculoskeletal disorders.

Considering the respective characteristics and adverse consequences of OP, SP, and OS, the aims of this study were to (1) compare the prevalence of three states of participants under different BMI and PBF levels, (2) explore the associations of BMI and PBF with bone mass and muscle quality (muscle mass and strength), and (3) elucidate the feasibility of the BMI and PBF as disease-identification tools. Our findings provide a valuable reference for clinicians to undertake primary screenings for musculoskeletal diseases in high-risk populations based on simple clinical indicators.

## 2. Materials and Methods

### 2.1. Research Ethics

This study was conducted in accordance with the Declaration of Helsinki and approved by the Ethics Committee of the First Affiliated Hospital of Sun Yat-sen University (protocol code: [2024]167; approved 22 March 2024). Due to the nature of this retrospective study and the preserved anonymity of patients, a waiver of informed consent was obtained.

### 2.2. Participants

**Figure 1:**
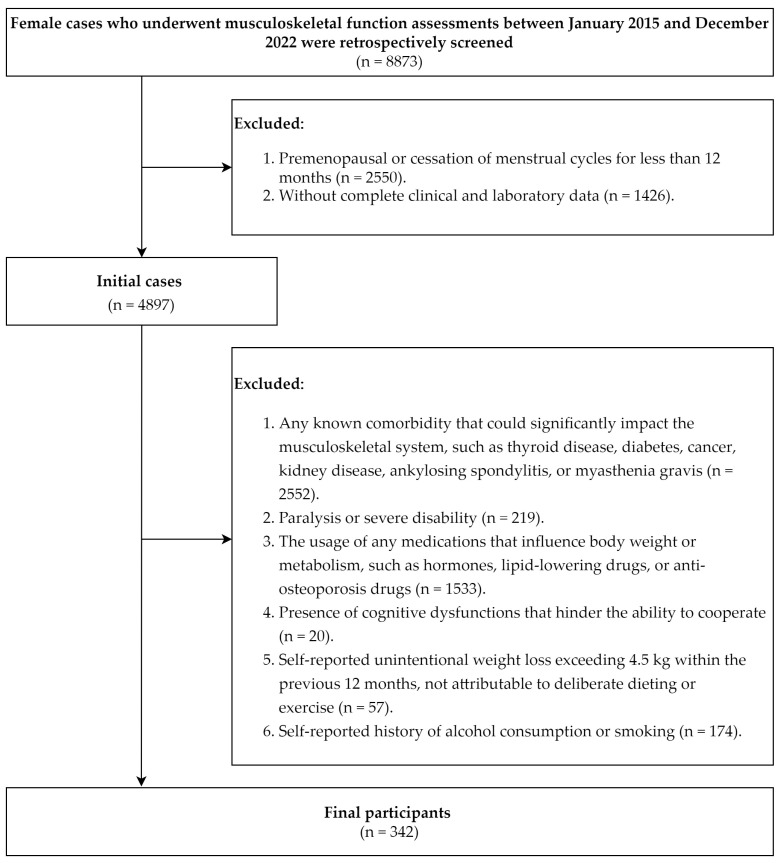
Flow diagram of participant inclusion.

This retrospective study collected electronic medical records of patients who underwent musculoskeletal function assessments at the First Affiliated Hospital of Sun Yat-sen University between January 2015 and December 2022. The inclusion criteria were as follows: (1) self-reported cessation of menstrual cycles for at least 12 months, and (2) complete clinical and laboratory data. Exclusion criteria included (1) any known comorbidity that could significantly impact the musculoskeletal system, such as thyroid disease, diabetes, cancer, kidney disease, ankylosing spondylitis, or myasthenia gravis; (2) paralysis or severe disability; (3) the usage of any medications that influence body weight or metabolism, such as hormones, lipid-lowering drugs, or anti-osteoporosis drugs; (4) the presence of cognitive dysfunctions that hinder one’s ability to cooperate; (5) self-reported unintentional weight loss exceeding 4.5 kg within the previous 12 months not attributable to deliberate dieting or exercise; and (6) self-reported history of alcohol consumption or smoking. After exclusion, 342 eligible participants were included in the final analysis (Figure 1).

### 2.3. Demographic Data

The general characteristics of the participants, including their age, menopausal duration, height, weight, and BMI, were collected. The menopausal duration was calculated by subtracting the age at the onset of menopause from the chronological age. The BMI was calculated as weight in kilograms divided by the square of the height in meters.

### 2.4. Laboratory Examination

Laboratory data were extracted from electronic medical records, including total serum protein, albumin, hemoglobin, calcium, phosphorus, alkaline phosphatase (ALP), total cholesterol, triglycerides, high-density lipoprotein cholesterol (HDL-C), low-density lipoprotein cholesterol (LDL-C), apolipoprotein A1 (ApoA1), polipoprotein B (ApoB), apolipoprotein E (ApoE), and lipoprotein α (Lpα) on admission.

### 2.5. Musculoskeletal Function Assessment

Quantitative data regarding body composition were acquired using a dual-energy X-ray absorptiometry (Lunar iDXA; GE Healthcare, Chicago, IL, USA). The lowest T-score among the lumbar spine, femoral neck, and total hip was used for the BMD assessment. The PBF was calculated by dividing the fat mass by the total body mass and multiplying by 100. The relative skeletal muscle index (RSMI) was used to evaluate the muscle mass, calculated as the appendicular skeletal muscle mass in kilograms divided by the height in meters squared [16]. The regional fat distribution parameters, such as android fat, gynoid fat, android-to-gynoid fat ratio, trunk-to-total body fat ratio, thigh-to-total body fat ratio, and appendicular-to-trunk fat ratio, were also recorded. Grip strength (GS) was used to evaluate the muscle strength, acquired using a handheld dynamometer equipped with strain gauge sensors (EH101, Xiangshan Inc., Zhongshan, China).

### 2.6. Diagnosis and Grouping

OP was diagnosed according to the World Health Organization criteria: T-score ≤ −2.5 for OP, between −2.5 and −1.0 for osteopenia, and >−1.0 for normality [17]. Based on the 2019 consensus of the Asian Working Group for Sarcopenia, females with an RSMI < 5.4 kg/m^2^ and a GS < 18 kg were considered as having SP [16].

The participants were divided into four groups based on the types of diseases: (1) OP alone group: participants who had OP alone but did not meet the diagnostic criteria for SP; (2) SP alone group: participants who had SP alone but did not meet the diagnostic criteria for osteopenia or OP; (3) OS group: participants who had both SP and either osteopenia or OP; and (4) control group: remaining participants.

### 2.7. Statistical Analysis

Statistical analysis was performed using IBM SPSS Statistics 22.0 software (IBM, Armonk, NY, USA). Continuous variables are presented as the mean (standard deviation). Categorical variables are expressed as frequencies (n) and percentages (%). First, participants were stratified according to the BMI quartiles (BMI_~Q1_ [≤25th percentile], BMI_Q1~Q3_ [25th to 75th percentile], and BMI_Q3~_ [≥75th percentile]) and PBF quartiles (PBF_~Q1_ [≤25th percentile], PBF_Q1~Q3_ [25th to 75th percentile], and PBF_Q3~_ [≥75th percentile]), respectively. Independent variables with statistical significance in both the BMI and PBF stratifications were screened by one-way ANOVA. A chi-square test was used to compare the disease prevalences between different stratifications. Second, a Pearson correlation analysis was used to analyze the associations between the BMI, PBF, and musculoskeletal function assessment parameters. Subsequently, logistic regression was used to explore the relationship between the BMI and PBF with the types of disease. Finally, receiver operating characteristics curves were plotted, and areas under the curve (AUCs) were calculated to evaluate the predictive accuracy of the BMI and PBF for diseases. Differences between the AUCs were compared using the DeLong test [18]. A two-tailed *p* < 0.05 was considered statistically significant and a Bonferroni-corrected *p*-value (0.05/3 = 0.017) was considered where applicable.

## 3. Results

### 3.1. General Characteristics

Based on the World Health Organization criteria and the 2019 consensus of the Asian Working Group for Sarcopenia, 26 participants (7.6%) were diagnosed with OP alone, 23 participants (6.7%) were diagnosed with SP alone, and 52 participants (15.2%) were defined as having OS.

Table 1 shows the clinical characteristics of participants under the BMI stratification. The differences in age and menopausal duration between the participants under different BMI stratifications were not statistically significant; however, statistically significant differences were observed in the parameters of the musculoskeletal function assessment. Specifically, participants with a high BMI stratification had a significantly higher RSMI and GS than participants with a low BMI stratification.

Table 2 shows the clinical characteristics of participants under PBF stratification. Different from BMI stratification, the menopausal duration of PBF_Q3~_ was significantly higher than that of PBF_Q1~Q3_. For the musculoskeletal function assessment parameters, participants in the PBF_~Q1_ had a statistically lower BMD, but the differences in the GS between the different PBF stratifications were not significant.

Anthropometric indicators, such as the BMD, RSMI, android fat, gynoid fat, android-to-gynoid fat ratio, trunk-to-total body fat ratio, thigh-to-total body fat ratio, and appendicular-to-trunk fat ratio—as well as laboratory indexes, such as hemoglobin, phosphorus, triglyceride, LDL-C, and ApoB—showed significant differences between the different stratifications of the BMI and PBF.

### 3.2. Prevalence of Diseases

**Figure 2:**
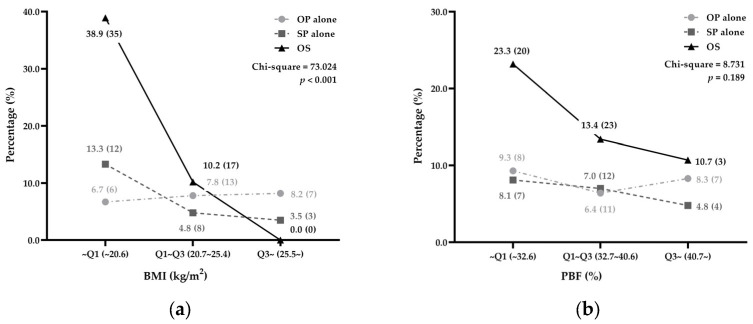
Prevalence of diseases. (**a**) BMI-stratified; (**b**) PBF-stratified. Data are expressed as % (n). BMI, body mass index; PBF, percent body fat; OP, osteoporosis; SP, sarcopenia; OS, osteosarcopenia.

As the levels of the BMI and PBF increased, the prevalence of SP alone and OS showed a consistent decline (Figure 2). However, with the increase in the BMI level, the prevalence of OP alone showed a sustained and slow upward trend (Figure 2a). In contrast, as the level of the PBF increased, the prevalence of OP alone showed a trend of first declining and then rising (Figure 2b). In the chi-square test, there was a statistically significant difference in the prevalence of the three states in the BMI stratification (chi-square = 73.024, *p* < 0.001), but not in the PBF stratification (chi-square = 8.731, *p* = 0.189).

Furthermore, a pairwise comparison found that there were statistically significant differences in the prevalence of three diseases between the BMI_~Q1_ and the BMI_Q1~Q3_ (chi-square = 41.229, Bonferroni-corrected *p* < 0.001) and between the BMI_~Q1_ and the BMI_Q3~_ (chi-square = 53.270, Bonferroni-corrected *p* < 0.001). However, the difference between the BMI_Q1~Q3_ and the BMI_Q3~_ was not statistically significant (chi-square = 9.713, Bonferroni-corrected *p* = 0.021). Figure S1 shows the prevalence of diseases in the substratification.

### 3.3. Correlations of BMI and PBF with Musculoskeletal Function Assessment Parameters

A significant positive correlation was observed between the BMI and musculoskeletal function assessment parameters, either before or after controlling for confounders, with the BMI and RSMI having the strongest correlation. However, the PBF was positively correlated with the BMD and RSMI in the simple correlation analysis, but the partial correlation analysis only showed a negative correlation between the PBF and RSMI (see details in Table 3).

### 3.4. Associations of BMI and PBF with the Types of Diseases

The BMI demonstrated a significant impact on the individuals with SP alone (OR = 0.592, 95% CI: 0.455–0.769) or OS (OR = 0.411, 95% CI: 0.319–0.529) when compared with the control group. Briefly, a higher BMI was found to be a significant protective factor against the risk of developing SP alone (*β* = –0.524) or OS (*β* = –0.890). The association between the PBF and OS revealed that a higher PBF was significantly associated with the greater prevalence of OS (OR = 1.404, 95% CI: 1.007–1.959), indicating that the PBF was a risk factor for OS. However, the BMI and PBF were not significantly associated with OP alone (see details in Table 4).

### 3.5. Discriminative Abilities of BMI and PBF for Diseases

The capacities of the BMI and PBF to discriminate participants with OP alone, SP alone, and OS was further evaluated, and the results are shown in Table 5. Neither the BMI nor the PBF could identify the participants with OP alone. The BMI, but not the PBF, showed a moderate diagnostic accuracy to identify participants with SP alone (AUC = 0.682), and both the BMI and PBF were effective in identifying participants with OS (AUC = 0.823 and AUC = 0.613, respectively). Notably, when the BMI cut-off value was set at 20.8 kg/m^2^, the BMI exhibited superior discriminative ability for participants with OS, where it achieved an AUC of 0.823, which was significantly greater than that of the PBF (*p* < 0.001, DeLong test).

## 4. Discussion

In this study, we investigated the relationship of the BMI and PBF with OP, SP, and OS in a cohort of 342 postmenopausal women in China. Additionally, we assessed the utility of the BMI and PBF as diagnostic tools for differentiating these conditions. Consistent with our hypotheses, the BMI demonstrated a positive correlation with the musculoskeletal function assessment parameters, like BMD, RSMI, and GS. However, after controlling for confounding variables, the PBF was found to be negatively associated with the RSMI only. Furthermore, the BMI exhibited superior diagnostic accuracy for participants with SP alone or OS compared with the PBF. These findings imply that the impacts and underlying mechanisms of the BMI and PBF on the musculoskeletal system in postmenopausal women may vary.

In humans, the mass of the skeletal muscle occupies 38–54% and 28–39% of the total body mass of men and women, respectively [19]. From youth to old age, the skeletal muscle mass decreases by an average of 40% [20], while body fat increases by over 50% [21]. A loss of lean mass and increase in fat mass are commonly observed in postmenopausal women and have been reported to be strongly associated with the development of OP or SP. Furthermore, an increased accumulation of body fat is considered a risk factor for accelerated aging and reduced life expectancy [22]. Adipose tissue is the main site for storing and mobilizing energy in response to metabolic demand. Post-menopause, adipose tissue is the main source of estrogen biosynthesis [23]. Adiponectin is a circulating adipokine secreted by mature adipocytes. Serum adiponectin may protect against SP via up-regulating the PI3K/AKT pathway, which promotes muscle protein synthesis and prevents muscle protein degradation [24]. However, excessive fat accumulation in skeletal muscles can impair insulin signaling and glucose intake [25]. Our recent study indicated that the abnormal lineage commitment of bone marrow mesenchymal stem cells is closely related to bone loss and fat accumulation in bone marrow during OP [26]. In this study, a high BMI was an independent protective factor for SP alone or OS, which is consistent with the findings of Yoo et al. [27]. The findings suggest that the BMI remains a partially effective indicator of lean mass reduction within the study population. Moreover, the detrimental impact of excessive adipose tissue accumulation on the musculoskeletal system may significantly surpass any potential benefits.

Although there is an association between the BMI and PBF with one or more musculoskeletal function assessment parameters, neither the BMI nor PBF proved effective in identifying patients with OP alone. These discrepancies can be attributed to several factors: according to the grouping criteria utilized in this study, a portion of individuals diagnosed with OP alone also exhibited normal RSMI levels, yet had not met the diagnostic criteria for SP. The significant correlation between the BMI and PBF and the RSMI may have reduced the effectiveness of both variables in identifying the participants with OP alone. Furthermore, several recent studies employed Mendelian randomization to estimate the causal effects of SP on OP, suggesting that SP may contribute to an accelerated decline in BMD at specific anatomical sites [28,29,30]. These SP-related changes in body composition are more likely to be reflected earlier in measures such as the BMI and PBF. In addition, the diagnosis of OP in our study was determined by the lowest T-score across three measurement sites. The influence of the BMI and PBF on the BMD may vary across different anatomical locations, a variation that is also evident in the differential response of these sites to various treatments [31].

Malnutrition is a recognized risk factor for OP or SP in the elderly [32,33]. As a coexisting state of low bone mass and low muscle quality, a cross-sectional study of older hospitalized patients pointed out that OS is associated with a higher degree of malnutrition than OP or SP alone [34]. In the elderly population, a low BMI or a low PBF largely indicates a state of nutritional deficiency. In this study, with the increase in the BMI and PBF, the overall prevalence of diseases decreased. This finding highlights the protective effect of improved nutrition on musculoskeletal health. It is noteworthy that the BMI_O1~Q3_PBF_O3~_ substratification included many individuals with normal weight but a high PBF, known as normal-weight obesity, which is often overlooked in clinical practice due to its hidden nature [35]. Accumulating evidence indicates that normal-weight obesity is associated with a significantly higher risk of developing metabolic syndrome and cardiometabolic dysfunction, as well as a higher mortality [35,36,37]. The high prevalence of diseases observed in the BMI_O1~Q3_PBF_O3~_ indicates that a comprehensive evaluation of musculoskeletal disorders necessitates the assessment of the PBF, even among individuals whose body weight is classified as normal according to the BMI criteria.

The utilization of radiographic methods like dual-energy X-ray absorptiometry or high-resolution quantitative CT to assess the regional fat distribution offers a more detailed understanding of alterations in the body composition compared with the whole-body assessments of the BMI or PBF. Different adipose tissue compartments are thought to have differing metabolic effects [38]. The enzymatic activity of aromatase, which is crucial for the synthesis of estradiol, exhibits a significantly higher level of gluteal fat than omental fat [11,39]. Regarding the android fat, gynoid fat, android-to-gynoid fat ratio, trunk-to-total body fat ratio, thigh-to-total body fat ratio, and appendicular-to-trunk fat ratio levels, our study found significant differences between the stratifications. In order to eliminate these confounding factors, we adjusted for these variables and found that with the addition of these covariables, the positive association between the PBF and RSMI flipped and became negative. This interesting finding emphasizes the prospect of further research on the association of the PBF and regional fat distribution with musculoskeletal diseases.

In our study, with the increase in the BMI and PBF, the levels of hemoglobin, phosphorus, triglyceride, LDL-C, and ApoB were also increased. Individuals who are malnourished or at risk of malnutrition often have a low hemoglobin level. A cross-sectional study from elderly residents in Australia showed that the incidence of anemia was the highest among SP patients (39%), followed by osteosarcopenic (34%), osteoporotic/penic (26%), and control (24%) patients [40]. Decreased phosphorus was considered to be associated with the increased BMD of the lumbar spine within one year of treatment with anti-osteoporosis drugs [31]. However, pathologically low levels of phosphorus have been reported to be significantly associated with a decline in hand grip strength [41]. ApoB is the primary apolipoprotein constituent of LDL-C and is important in the transportation and metabolic processes of LDL-C [42]. Lipid dysmetabolism leads to a reduction in HDL-C and ApoA levels and an increase in LDL-C, insulin, triglyceride, and ApoB levels [43]. A comparative study from Kobayashi et al. found that in men, the PBF was a stronger determinant of LDL-C and triglyceride than the BMI, while in women, the BMI was a stronger determinant of HDL-C than the PBF [44]. Our results provide a basis for further pursuing the development of a serum-metabolomics-based biomarker for musculoskeletal disorders.

This study had some limitations. First, the BMI and PBF are influenced by variables such as race, sex, and age. The cut-off values proposed in this study may be applicable solely to specific populations. Second, the retrospective design and limited sample size constrained the statistical power. Third, the current diagnostic criteria for OS are inconsistent. In alignment with this study, the majority of research defined OS as the co-existing state of SP and either osteopenia or OP. However, other studies defined OS as the co-existing state of SP and OP [5,45].

## 5. Conclusions

To the best of our knowledge, this is one of the few studies that evaluated the associations of body mass index and percent body fat with osteoporosis, sarcopenia, and osteosarcopenia. In postmenopausal individuals, a higher BMI and lower PBF are associated with a lower prevalence of the co-existence of bone loss and muscle degeneration. Considering that the BMI and PBF can be easily measured by healthcare professionals, researchers, and even individuals at home, they remain an effective and low-cost tool for the initial assessments of musculoskeletal disorders, particularly in settings with limited resources.

## Figures and Tables

**Table 1:** Clinical characteristics of participants under BMI stratification (n = 342).

Variable	BMI (kg/m^2^)				
	~Q1 (~20.6) (n = 90)	Q1~Q3 (20.7~25.4) (n = 167)	Q3~ (25.5~) (n = 85)	*F*	*p*
Age (y)	64.1 (12.3)	62.2 (9.7)	65.2 (10.7)	2.534	0.081
Menopausal duration (y)	14.0 (11.5)	11.7 (9.2)	14.5 (10.0)	2.690	0.069
Musculoskeletal function assessment					
BMD (T-score)	−1.6 (1.3)	−0.9 (1.3)	−0.4 (1.3)	20.728	**<0.001**
RSMI (kg/m^2^)	5.29 (0.66) ^bc^	5.90 (0.59) ^ac^	6.67 (0.73) ^ab^	100.520	**<0.001**
GS (kg)	17 (8) ^bc^	22 (8) ^ac^	24 (7) ^ab^	22.230	**<0.001**
Body composition					
Android fat (%)	29.7 (9.8) ^bc^	40.3 (7.5) ^ac^	48.3 (5.6) ^ab^	125.781	**<0.001**
Gynoid fat (%)	33.2 (6.1) ^bc^	37.7 (4.8) ^ac^	41.4 (5.3) ^ab^	52.740	**<0.001**
Android-to-gynoid fat ratio	0.89 (0.23) ^bc^	1.07 (0.19) ^ac^	1.18 (0.14) ^ab^	51.443	**<0.001**
Trunk-to-total body fat ratio	0.49 (0.06) ^bc^	0.54 (0.06) ^ac^	0.57 (0.04) ^ab^	48.133	**<0.001**
Thigh-to-total body fat ratio	0.33 (0.05) ^bc^	0.30 (0.05) ^ac^	0.28 (0.04) ^ab^	24.402	**<0.001**
Appendicular-to-trunk fat ratio	0.96 (0.26) ^bc^	0.80 (0.20) ^ac^	0.71 (0.14) ^ab^	33.228	**<0.001**
Laboratory examination					
Total protein (g/L)	67.0 (6.7)	67.8 (6.2)	67.5 (6.8)	0.491	0.613
Albumin (g/L)	39.1 (4.9)	39.8 (4.3)	39.8 (4.2)	0.737	0.479
Hemoglobin (g/L)	122 (16) ^bc^	127 (12) ^ac^	132 (12) ^ab^	11.597	**<0.001**
Calcium (mmol/L)	2.26 (0.14)	2.29 (0.15)	2.29 (0.10)	1.766	0.173
Phosphorus (mmol/L)	1.12 (0.22) ^bc^	1.18 (0.19) ^a^	1.20 (0.18) ^a^	4.568	**0.011**
ALP (U/L)	82 (39)	81 (33) ^c^	73 (19) ^b^	2.305	0.101
Total cholesterol (mmol/L)	4.85 (1.20) ^c^	5.07 (1.08)	5.26 (1.17) ^a^	2.865	0.058
Triglyceride (mmol/L)	1.26 (0.70) ^c^	1.40 (0.76) ^c^	1.90 (1.52) ^ab^	10.153	**<0.001**
HDL-C (mmol/L)	1.41 (0.40)	1.41 (0.37)	1.32 (0.29)	1.830	0.162
LDL-C (mmol/L)	2.86 (0.81) ^bc^	3.08 (0.79) ^a^	3.23 (0.86) ^a^	4.741	**0.009**
ApoA1 (g/L)	1.42 (0.31)	1.45 (0.30)	1.42 (0.28)	0.547	0.579
ApoB (g/L)	0.82 (0.24) ^c^	0.86 (0.22) ^c^	0.94 (0.26) ^ab^	5.597	**0.004**
ApoA1-to-ApoB ratio	1.87 (0.65)	1.80 (0.67)	1.68 (0.73)	1.897	0.152
ApoE (mg/L)	42 (13) ^c^	44 (13)	46 (13) ^a^	2.443	0.088
Lpα (mg/L)	191 (226)	240 (260)	264 (324)	1.739	0.177

Data are shown as mean (standard deviation). All *p*-values were calculated with the one-way ANOVA. Significant *p*-values are presented in bold. ^a^ Compared with ~Q1, *p* < 0.05; ^b^ compared with Q1~Q3, *p* < 0.05; ^c^ compared with Q3~, *p* < 0.05. BMI, body mass index; BMD, bone mineral density; RSMI, relative skeletal muscle index; GS, grip strength; ALP, alkaline phosphatase; HDL-C, high-density lipoprotein cholesterol; LDL-C, low-density lipoprotein cholesterol; Apo, apolipoprotein; Lp, lipoprotein.

**Table 2:** Clinical characteristics of participants under PBF stratification (n = 342).

Variable	PBF (%)				
	~Q1 (~32.6) (n = 86)	Q1~Q3 (32.7~40.6) (n = 172)	Q3~ (40.7~) (n = 84)	*F*	*p*
Age (y)	63.1 (12.2)	62.4 (9.9) ^c^	65.8 (10.5) ^b^	2.956	0.053
Menopausal duration (y)	12.8 (11.1)	11.9 (9.7) ^c^	15.5 (9.6) ^b^	3.741	**0.025**
Musculoskeletal function assessment					
BMD (T-score)	−1.4 (1.3) ^bc^	−0.9 (1.4) ^a^	−0.8 (1.4) ^a^	5.256	**0.006**
RSMI (kg/m^2^)	5.61 (0.77) ^bc^	5.97 (0.73) ^ac^	6.18 (0.91) ^ab^	11.460	**<0.001**
GS (kg)	20 (9)	22 (8)	21 (7)	0.892	0.411
Body composition					
Android fat (%)	25.9 (7.37) ^bc^	41.1 (4.59) ^ac^	50.2 (4.28) ^ab^	453.816	**<0.001**
Gynoid fat (%)	30.6 (4.6) ^bc^	37.9 (3.7) ^ac^	43.5 (3.9) ^ab^	225.551	**<0.001**
Android-to-gynoid fat ratio	0.85 (0.24) ^bc^	1.10 (0.17) ^ac^	1.16 (0.13) ^ab^	71.313	**<0.001**
Trunk-to-total body fat ratio	0.47 (0.07) ^bc^	0.54 (0.05) ^ac^	0.57 (0.04) ^ab^	77.725	**<0.001**
Thigh-to-total body fat ratio	0.34 (0.06) ^bc^	0.30 (0.05) ^ac^	0.28 (0.04) ^ab^	32.632	**<0.001**
Appendicular-to-trunk fat ratio	1.01 (0.29) ^bc^	0.78 (0.16) ^ac^	0.70 (0.13) ^ab^	56.945	**<0.001**
Laboratory examination					
Total protein (g/L)	66.5 (7.1)	68.1 (6.0)	67.3 (6.8)	1.946	0.144
Albumin (g/L)	38.8 (5.1)	40.3 (3.9)	39.1 (4.4)	4.262	**0.015**
Hemoglobin (g/L)	120 (15) ^bc^	128 (12) ^a^	130 (12) ^a^	14.922	**<0.001**
Calcium (mmol/L)	2.26 (0.15)	2.28 (0.14)	2.30 (0.13)	1.233	0.293
Phosphorus (mmol/L)	1.11 (0.22) ^bc^	1.17 (0.17) ^a^	1.21 (0.21) ^a^	5.644	**0.004**
ALP (U/L)	81 (33)	81 (35)	74 (24)	1.580	0.207
Total cholesterol (mmol/L)	4.69 (1.11) ^bc^	5.16 (1.12) ^a^	5.23 (1.15) ^a^	6.450	**0.002**
Triglyceride (mmol/L)	1.18 (0.55) ^bc^	1.56 (1.23) ^a^	1.66 (0.82) ^a^	5.862	**0.003**
HDL-C (mmol/L)	1.42 (0.41)	1.40 (0.35)	1.34 (0.33)	1.249	0.288
LDL-C (mmol/L)	2.75 (0.73) ^bc^	3.13 (0.82) ^a^	3.24 (0.84) ^a^	9.110	**<0.001**
ApoA1 (g/L)	1.41 (0.31)	1.45 (0.29)	1.43 (0.30)	0.525	0.592
ApoB (g/L)	0.78 (0.21) ^bc^	0.88 (0.23) ^ac^	0.94 (0.25) ^ab^	11.543	**<0.001**
ApoA1-to-ApoB ratio	1.94 (0.66) ^c^	1.79 (0.70)	1.64 (0.64) ^a^	4.189	**0.016**
ApoE (mg/L)	41 (12) ^bc^	45 (14) ^a^	46 (13) ^a^	3.010	0.051
Lpα (mg/L)	203 (226)	237 (267)	257 (270)	0.876	0.417

Data are shown as mean (standard deviation). All *p*-values were calculated with the one-way ANOVA. Significant *p*-values are presented in bold. ^a^ Compared with ~Q1, *p* < 0.05; ^b^ compared with Q1~Q3, *p* < 0.05; ^c^ compared with Q3~, *p* < 0.05. PBF, percent body fat; BMD, bone mineral density; RSMI, relative skeletal muscle index; GS, grip strength; ALP, alkaline phosphatase; HDL-C, high-density lipoprotein cholesterol; LDL-C, low-density lipoprotein cholesterol; Apo, apolipoprotein; Lp, lipoprotein.

**Table 3:** Correlations of BMI and PBF with musculoskeletal function assessment parameters.

Variable	BMD (T-Score)		RSMI (kg/m^2^)		GS (kg)	
	Simple	Partial	Simple	Partial	Simple	Partial
BMI (kg/m^2^)	0.373 **	0.378 **	0.663 **	0.666 **	0.354 **	0.415 **
PBF (%)	0.174 **	0.070	0.265 **	−0.134 *	0.058	−0.099

Data are shown as Pearson correlation coefficient (r). All *p*-values were calculated with the Pearson correlation or covariate-adjusted partial correlation test. The covariates include android fat, gynoid fat, android-to-gynoid fat ratio, trunk-to-total body fat ratio, thigh-to-total body fat ratio, appendicular-to-trunk fat ratio, hemoglobin, phosphorus, triglyceride, LDL-C, and ApoB. * *p* < 0.05; ** *p* < 0.01. BMI, body mass index; PBF, percent body fat; BMD, bone mineral density; RSMI, relative skeletal muscle index; GS, grip strength; LDL-C, low-density lipoprotein cholesterol; Apo, apolipoprotein.

**Table 4:** Associations of BMI and PBF with the types of disease in multivariate logistic regression.

Variable	Unadjusted			Fully Adjusted		
	OR	95% CI	*p*	OR	95% CI	*p*
OP alone (n = 26)						
BMI	0.921	0.811–1.045	0.201	0.851	0.687–1.053	0.138
PBF	0.654	0.925–1.050	0.314	0.970	0.625–1.504	0.891
SP alone (n = 23)						
BMI	0.751	0.645–0.876	**<0.001**	0.592	0.455–0.769	**<0.001**
PBF	0.981	0.918–1.048	0.570	1.502	0.928–2.432	0.098
OS (n = 52)						
BMI	0.610	0.532–0.700	**<0.001**	0.411	0.319–0.529	**<0.001**
PBF	0.928	0.887–0.971	**0.001**	1.404	1.007–1.959	**0.046**

The control group was used as the reference (n = 241). Significant *p*-values are presented in bold. Values in the fully adjusted categories were adjusted for android fat, gynoid fat, android-to-gynoid fat ratio, trunk-to-total body fat ratio, thigh-to-total body fat ratio, appendicular-to-trunk fat ratio, hemoglobin, phosphorus, triglyceride, LDL-C, and ApoB. BMI, body mass index; PBF, percent body fat; OP, osteoporosis; SP, sarcopenia; OS, osteosarcopenia; LDL-C, low-density lipoprotein cholesterol; Apo, apolipoprotein.

**Table 5:** Performance characteristics of the BMI and the PBF for distinguishing diseases.

Variable	AUC (95% CI)	*p*	Cut-Off Value	Sensitivity (%, 95% CI)	Specificity (%, 95% CI)
OP alone (n = 26)					
BMI	0.508 (0.454–0.563)	0.887	21.5	73.1 (52.2–88.4)	34.8 (29.6–40.3)
PBF	0.502 (0.448–0.557)	0.973	44.9	19.2 (6.6–39.4)	95.3 (92.3–97.3)
DeLong test		0.895			
SP alone (n = 23)					
BMI	0.682 (0.629–0.731)	**0.005**	21.4	69.6 (47.1–86.8)	69.0 (63.6–74.0)
PBF	0.523 (0.649–0.577)	0.702	33.2	43.5 (23.2–65.5)	72.7 (67.5–77.5)
DeLong test		**<0.001**			
OS (n = 52)					
BMI	0.823 (0.778–0.862)	**<0.001**	20.8	69.2 (54.9–81.3)	79.3 (74.2–83.8)
PBF	0.613 (0.559–0.665)	**0.013**	35.9	65.4 (50.9–78.0)	58.3 (52.4–64.0)
DeLong test		**<0.001**			

The control group was used as the reference (n = 241). Significant *p*-values are presented in bold. BMI, body mass index; PBF, percent body fat; OP, osteoporosis; SP, sarcopenia; OS, osteosarcopenia; AUC, area under the curve.

## Data Availability

The data presented in this study are available on request from the corresponding author. The data are not publicly available due to privacy of the study participants.

## Supplementary Materials

The following supporting information can be downloaded from https://www.mdpi.com/article/10.3390/healthcare13010028/s1, Figure S1: Prevalence of diseases in substratification.
