# Supplementary material for: Associations of Body Mass Index and Percent Body Fat with Osteoporosis, Sarcopenia, and Osteosarcopenia: A Retrospective Study Based on Postmenopausal Women in China
Source: Healthcare (Basel). 2024 Dec 26;13(1):28. doi: 10.3390/healthcare13010028 (PMC11719479; doi:10.3390/healthcare13010028)
Supplement: Supplementary file 1 [file healthcare-13-00028-s001.zip › healthcare-3363529-supplementary.pdf]

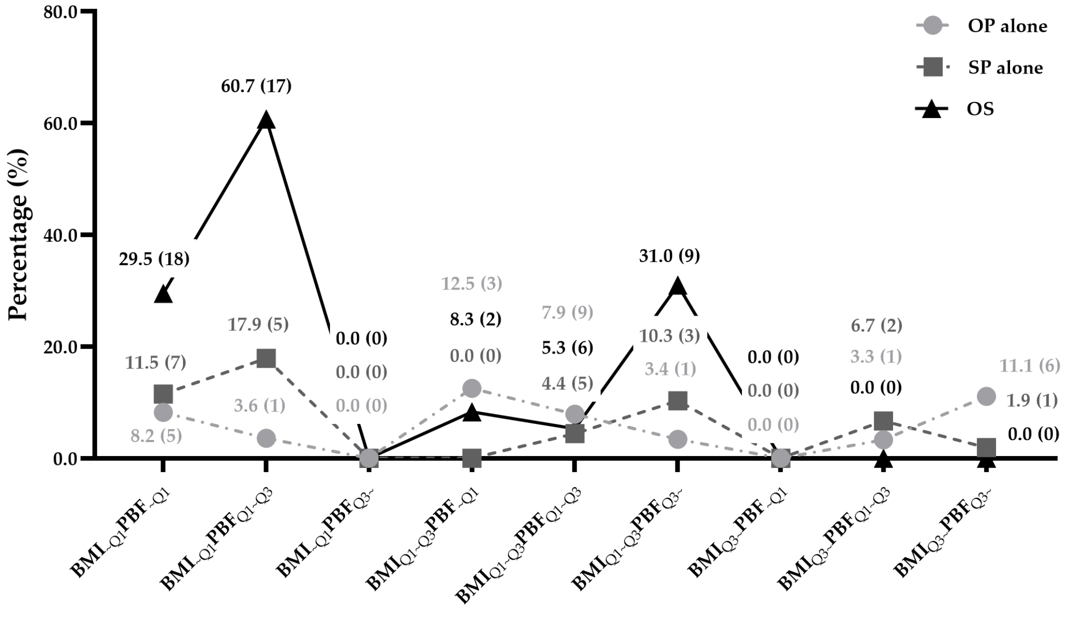

Figure S1. Prevalence of diseases in substratification. Data are expressed as % (n). BMI, body mass index; PBF, percent body fat; OP, osteoporosis; SP, sarcopenia; OS, osteosarcopenia.
